# Comparative microbiome analysis of beef cattle, the feedyard environment, and airborne particulate matter as a function of probiotic and antibiotic use, and change in pen environment

**DOI:** 10.3389/fmicb.2024.1348171

**Published:** 2024-02-08

**Authors:** A. H. Strickland, S. A. Murray, J. Vinasco, B. W. Auvermann, K. J. Bush, J. E. Sawyer, H. M. Scott, K. N. Norman

**Affiliations:** ^1^Department of Veterinary Integrative Biosciences, Texas A&M University, College Station, TX, United States; ^2^Department of Veterinary Pathobiology, Texas A&M University, College Station, TX, United States; ^3^Texas A&M AgriLife Research and Extension Center at Amarillo, Amarillo, TX, United States; ^4^Department of Animal Sciences, Texas A&M University, College Station, TX, United States

**Keywords:** environmental microbiome, fecal microbiome, particulate matter, antibiotic alternatives, antimicrobial resistance (AMR)

## Abstract

**Introduction:**

Intensive beef cattle production systems are frequently implicated as a source of bacteria that can be transferred to nearby humans and animals via effluent water, manure used as fertilizer, or airborne particulate matter. It is crucial to understand microbial population dynamics due to manure pack desiccation, antibiotic usage, and antibiotic alternatives within beef cattle and their associated feedyard environment. Understanding how bacterial communities change in the presence of antibiotics can also improve management practices for reducing the spread of foodborne bacteria.

**Methods:**

In this study, we aimed to compare the microbiomes within cattle feces, the feedyard environment and artificially produced airborne particulate matter as a function of pen change and treatment with tylosin or probiotics. We utilized 16S rRNA sequencing to compare bacterial communities among sample types, study days, and treatment groups.

**Results:**

Bacterial community diversity varied as a function of sampling day and pen change (old or new) within fecal and manure pack samples. Manure pack samples from old pens and new pens contained diverse communities of bacteria on days 0 and 84; however, by day 119 of the study these taxonomic differences were less evident. Particulate matter samples exhibited significant differences in community diversity and predominant bacterial taxa compared to the manure pack they originated from. Treatment with tylosin did not meaningfully impact bacterial communities among fecal, environmental, or particulate matter samples; however, minor differences in bacterial community structure were observed in feces from cattle treated with probiotics.

**Discussion:**

This study was the first to characterize and compare microbial communities within feces, manure pack, and airborne particulate matter from the same location and as a function of tylosin and probiotic treatment, and pen change. Although fecal and environmental samples are commonly used in research studies and other monitoring programs to infer public health risk of bacteria and antimicrobial resistance determinants from feedyard environments, our study suggests that these samples may not be appropriate to infer public health risk associated with airborne particulate matter.

## 1 Introduction

The increasing demand to produce meat products for human consumption has led to high volume and fast turnover practices in beef cattle production systems. The finishing period of feedyard cattle consequently puts animals at a higher risk for liver abscesses, reduced weight gain, and poor feed efficiency (Nagaraja and Lechtenberg, 2007; Ban and Guan, 2021). Along with the intense nature of the finishing period, bacterial infectious diseases are also responsible for negative animal performance, health risks, and welfare (Nagaraja and Lechtenberg, 2007; Brown and Lawrence, 2010; Huebner et al., 2019). The use of antimicrobials is a common on-farm practice for combatting both liver abscesses and infectious bacterial disease. For example, tylosin is a bacteriostatic antibiotic within the macrolide class that is frequently administered in feed to reduce the occurrence of liver abscesses in cattle (Huebner et al., 2019; Weinroth et al., 2019; Murray et al., 2020). Although tylosin is shown to be effective in most cases, the mechanism by which liver abscesses are reduced is not fully understood and treatment is not always associated with an absence of liver abscesses within individual cattle (Weinroth et al., 2019). Administration of tylosin has been associated with co-selection for bacterial resistance to the entire macrolide class of 14-, 15-, and 16- membered ring molecules (Marshall and Levy, 2011; Beukers et al., 2015); these include multiple antibiotics that are classified by the World Health Organization (WHO) as critically important for human health (World Health Organization [WHO], 2017). Tylosin administration may promote carriage of antimicrobial resistance (AMR) determinants in cattle, which may increase the risk of human infections associated with AMR pathogens from beef food products and the environment (Noyes et al., 2016; Huebner et al., 2019; Weinroth et al., 2019, 2022). There is a critical need to find alternative therapies that provide the same benefits of antibiotics without the risk of AMR selection and propagation in food animal production systems.

Microbial probiotics and fermentation products have been investigated as alternatives to antibiotics to improve both animal health and performance characteristics in beef cattle (McAllister et al., 2011; Huebner et al., 2019; Ban and Guan, 2021). These products are reported to be associated with reduced shedding of pathogenic bacteria, increased average daily gain, increased feed efficiency, enhanced fiber digestion and an overall enhancement of the gastrointestinal microbiome and animal health (McAllister et al., 2011; Huebner et al., 2019; Ban and Guan, 2021). *Saccharomyces cerevisiae* fermentation products (SCFP) have been shown to reduce the incidence of liver abscesses, and improve growth performance and carcass characteristics in finished cattle (Wagner et al., 2016). Additionally, *Enterococcus* species of gram-positive bacteria are commonly included in commercially available probiotics for beef cattle production (Amachawadi et al., 2018; Shridhar et al., 2022); this is due to the bacteria’s favorable metabolism, competitive exclusion, lack of virulence genes in probiotic strains, and ability to survive both within the cattle gastrointestinal tract and the feedyard environment (Amachawadi et al., 2018; Murray et al., 2020, 2022; Shridhar et al., 2022); however, not all studies highlight these findings. In one study by Huebner et al. (2019), there were no positive effects shown in liver abscess reduction when using SCFP. Similarly, researchers did not reveal a positive effect of administering SCFP on performance characteristics in dairy calves (Titi et al., 2008) or finished beef cattle (Geng et al., 2016). Interestingly, one group of researchers administered a probiotic containing *Enterococcus faecium* and *Saccharomyces cerevisiae* in feed and were able to isolate the probiotic strain from the environment over 100 days after the trial began (Murray et al., 2020). Researchers suggest that an *Enterococcus* probiotic may exhibit “faecal-environmental-oral cycling” which may enhance the persistence of the product (Murray et al., 2020). Given these findings, there remains a need for investigating the ability of probiotics to persist and elicit an effect in the feedyard environment. Furthermore, elucidating and characterizing the bacterial communities affected by both antibiotics and antibiotic alternatives is important for both human and animal health.

It is crucial to understand the effects of antimicrobials on the microbiome of beef cattle and their associated environment for best management practices. In one study by Adeyemi et al. (2020), the administration of a *Saccharomyces cerevisiae*-based direct-fed microbial was associated with significant alterations in the bacterial communities within cattle feces. Contrastingly, another research team found no differences in fecal bacterial communities between SCFP treated and untreated cattle (Huebner et al., 2019). Few studies have investigated the effects of an *Enterococcus faecium/Saccharomyces cerevisiae* probiotic on the microbiomes within individual cattle and in their feedyard environment; however, there remains a need to increase our understanding of the effects of concurrent antibiotic and probiotic administration on bacterial communities in finisher beef cattle and the feedyard environment. Multiple researchers have characterized the effects of antibiotic administration on the bacterial communities within individual cattle and the environment (Thomas et al., 2017; Doster et al., 2018; Weinroth et al., 2019). These researchers showed that geographic location of the feedyard was more associated with differing microbial communities than antibiotic administration. These findings suggest that environmental factors and management practices may play a greater role in influencing microbiomes in cattle than commonly administered antimicrobials (Doster et al., 2018; Weinroth et al., 2019). Other studies support this rationale, revealing that animals from different environments and production facilities exhibit dissimilar microbial communities (Huebner et al., 2019). In this way, improving environmental and management factors may more effectively reduce the spread of bacteria and AMR from food animal production systems.

The cattle feedyard environment has consistently been shown to harbor determinants of AMR and has been implicated as a reservoir capable of spreading AMR to nearby animals and humans via effluent water, airborne particulate matter and manure used as fertilizer, the impacts of which have not been fully elucidated (Guo et al., 2011; Bonifacio et al., 2012; Noyes et al., 2016; Wooten et al., 2019). The generation and emission of particulate matter from the feedyard environment is of particular importance in the High Plains region of Texas where a large proportion of U.S. beef cattle finishing takes place. This region experiences prolonged dry periods and a hot, windy climate, promoting the desiccation of the manure pack into fine particulate matter that can readily become airborne and travel outside of the feedyard production system under certain atmospheric conditions (Bonifacio et al., 2012; McEachran et al., 2015). Airborne particulate matter can be visually appreciated near feedyard production systems especially around dusk when still air, increased animal activity, decreased pen surface moisture, and increasing boundary-layer stability contributes to increased particulate matter concentrations near ground level (Urso et al., 2021). Airborne particulate matter originating from the cattle feedyard environment has been shown to transfer viable bacteria (Zaheer et al., 2019), AMR determinants (McEachran et al., 2015; Zaheer et al., 2019), antibiotics (Wooten et al., 2019; Zaheer et al., 2019), and growth promoting hormones (Blackwell et al., 2015), to surrounding environments; however, little emphasis has been placed to characterize the bacterial communities present within such particulate matter as a function of antibiotic alternatives. Increased resolution of bacterial population dynamics within beef cattle, the feedyard environment, and airborne particulate matter in response to commonly administered therapies is an essential part of assessing their utility and safety in finisher beef production.

The objective of our study was to characterize and compare the microbiomes in cattle feces and the feedyard environment as a function of tylosin and/or commercial probiotic treatment, and due to pen environment change. Additionally, we aimed to artificially model the transition of environmental manure pack desiccation to particulate matter to better characterize and simulate the metagenomic changes experienced in the cattle feedyard environment over time. This objective was aimed at understanding bacterial population dynamics and determining the risk of airborne particulate matter originating from the cattle feedyard environment for human health. While a number of studies have investigated the effects of SCFP and probiotics on the fecal microbiomes in beef cattle, there remains a need to increase our understanding of the effects of concurrent antibiotic and probiotic administration in finisher beef cattle on the bacterial communities present within environmental manure pack and particulate matter samples.

## 2 Materials and methods

### 2.1 Experimental design

A 2 × 2 × 2 full factorial and longitudinal controlled trial was conducted at Texas A&M AgriLife Research experimental feedyard in McGregor, TX during the second trial replicate of a longitudinal study published by Murray et al. (2020, 2022), which utilized 96 finisher steers randomly assigned to 8 pens for the first 84 days of the trial. Pen-level treatment groups included: (1) tylosin in feed, (2) probiotic in feed, (3) tylosin and probiotic in feed, or (4) untreated controls. On day 84, pens 1−8 were split in half, and six of the twelve steers in each pen were randomly assigned to newly constructed pens (pens 9−16). Pens 1−8 are hereafter referred to as old pens and pens 9−16 are hereafter referred to as new pens. New pens were manufactured for the purpose of this study and initially had never housed cattle previously administered antibiotics. Free range cows and calves on adjacent pastures were allowed access to the new pens for 4 weeks prior to the start of the study by Murray et al. (2020, 2022) to establish an initial manure pack and microbial composition baseline representative of bovine feces. Animal usage in this study underwent ethical consideration and approval by the Agriculture Animal Care and Use Committee (AACUC AUP #2015-026A).

Steers received tylosin (Tylan, Elanco, Greenfield, IN) in feed at 7.3 g/tonne for the first 84 days of the trial and subsequently went through a voluntary wash out period for the remainder of the study prior to slaughter. A commercially available probiotic containing 1.3 × 10^7^ CFU/g of both *Enterococcus faecium* (ST296) and *Saccharomyces cerevisiae* (Tri-Lution, Agri-King, Fulton, IL) was administered to steers in feed at 824.5 g/tonne for the entire 119 days of the trial. All personnel were blinded to treatment groups, except for feedyard employees who administered the treatments via the feed. Detailed methods including cattle diets, husbandry practices, and pen management have been described previously (Murray et al., 2020, 2022).

### 2.2 Sample collection and processing

Individual fecal grab sampling per rectum was performed for all steers (*n* = 96) using a new rectal palpation sleeve for each animal on days 0, 84, and 119 of the study for a total of 288 fecal samples. Manure pack environmental samples were taken at the pen level (*n* = 16) along a diagonal transect at 6 sites per pen (approximately 25 g per site) on days 0, 84, and 119 of the study using a shovel that was cleaned and sterilized with alcohol between pens for a total of 48 manure pack samples. Day 0 was the first day of the study and sampling occurred prior to cattle entering the study pens. No treatments had been administered to any cattle at that point in time. Manure pack samples from pens 9−16 on day 84 were taken while cattle were being divided into old versus new pens and prior to placement in the new pens. Day 119 was the last day of sampling before steers were sent to slaughter and served as the end point of the study. Therefore, fecal and manure pack samples were collected on days 0, 84, and 119 of the study. Immediately after collection, all samples were transported to a microbiological laboratory for further processing at Texas A&M University in College Station, TX. Upon arrival to the laboratory, fecal and manure pack samples were stored at 4°C overnight, then transferred to 5 ml tubes for preservation at −80°C until further use.

Day 84 manure pack (MP) samples were further processed in series into two additional sample types – dried/milled (DM) samples and particulate matter (PM) samples - to model the microbiome changes that naturally occur in the cattle feedyard environment over time as a function of desiccation and aerosolization to particulate matter. MP samples from each pen were kept in 102L bins and dried in the Texas A&M AgriLife Research and Extension biomass drying facility in Bushland, TX for 2 weeks. MP was then ground with a 7hp hammer mill using a 3.2 mm screen (Texas A&M AgriLife Research and Extension, Bushland, TX). The mill was cleaned and sterilized with alcohol between samples. An aliquot of each DM sample was taken immediately following milling in 50 ml conical tubes and sent to the laboratory at Texas A&M University in College Station, TX. The remaining DM samples were then used to create aerosolized PM in the downstream section of a 61 cm X 61 cm X 244cm hoof action simulator test chamber (Texas A&M AgriLife Research and Extension). 50 ml of each DM sample was inserted into the airstream from the top of the test chamber and the aerosolized particles were captured on 8 × 10in 1 mm glass fiber filters (Danaher Corp, Washington, D.C.) positioned 1.5 m from the insertion point. Each filter was exposed to aerosolized particles for 1 min in the test chamber at a flow rate of 160CFM to collect the PM sample. The test chamber was cleaned and sterilized with alcohol between samples. Filters containing the PM samples were wrapped in foil, placed in individually labeled bags, and maintained at 4°C until further processing.

Fecal samples were pooled for community DNA extraction to include three fecal samples from the same animals within each pen for days 0, 84, and 119, resulting in a total of 96 pooled fecal samples balanced across pen, day, and treatment. Samples were thoroughly vortexed prior to subsampling for pooling and subsequent DNA extraction such that a representative sub-sample was obtained. Manure pack and DM samples were homogenized using a Tissue Lyser II^®^ (Qiagen, Valencia, CA) with 25 cycles per second for 10 min prior to subsampling for community DNA extraction.

### 2.3 Molecular methods

Community DNA extraction from pooled fecal, manure pack, and Day 84 samples was performed using the QIAGEN DNeasy^®^ PowerSoil^®^ Pro kit (Qiagen, Valencia, CA) per the manufacturer’s protocol within the automated QIAcube robot. Library preparation was conducted using Nextera XT Index Kits (Illumina, Inc., San Diego, CA) and Mastercycler^®^ Thermal Cycler (Eppendorf, Enfield, CT). Amplicon primers 341F (5’-CCTACGGGNGGCWGCAG-3’) and 785R (5′-GACTACHVGGGTATCTA ATCC-3′) with index adapters were utilized to specifically target and amplify the hypervariable V3/V4 region of the bacterial 16S rRNA gene (Klindworth et al., 2013). Library validation was performed on the Fragment Analyzer System (Agilent Technologies, Inc., Santa Clara, CA) prior to paired-end amplicon sequencing (2 × 300) of the 16S rRNA gene using MiSeq reagent v3 kits and the Illumina MiSeq platform (Illumina, Inc.) per manufacturer’s protocol. The 16S rRNA amplicon sequence data can be found in the NCBI database under BioProject accession number: PRJNA595617.

### 2.4 Sequence data analysis and bioinformatics

Raw fastq files that underwent primer removal and demultiplexing were utilized for 16S rRNA amplicon sequencing analysis using the QIIME 2 pipeline (version qiime2-2022.2) (Bolyen et al., 2019) within the Terra cluster on the Texas A&M High Performance Research Computing (HPRC) system (College Station, TX). Data were divided into three groups: pooled feces (Days 0, 84, and 119), manure pack pen samples (Days 0, 84, and 119) and Day 84 manure pack samples that underwent further serial processing (MP to DM, and then to PM). These sample groups were analyzed independently for greater resolution of variation among study days and sample types. Using the DADA2 plugin (Callahan et al., 2016), single-end reads were assigned amplicon sequence variants (ASVs), noise and chimeras were filtered from all samples and quality trimming was performed at a quality score threshold of 20%. Due to poor quality of reverse reads and inability of sequences to overlap during DADA2 without sacrificing sequence quality, the reverse reads were excluded from further analysis for all samples. The latest version of the SILVA classifier was utilized to assign taxonomic classification to sequences (silva-138-99-nb-classifier.qza) (Quast et al., 2013). ASV data, feature tables, and metadata files from QIIME 2 were transferred to RStudio for further bacterial microbiome characterization and statistical analysis using “metagenomeSeq” (Paulson et al., 2013a), “metagMisc” (Mikryukov, 2023), “phyloseq”(McMurdie and Holmes, 2013), and “vegan” packages within the RStudio platform (Oksanen et al., 2022). Data were filtered to remove any ASV of non-bacterial classification and were normalized using the cumulative sum scaling method as described by Paulson et al. (2013b) to adjust for variation in sampling depth across samples.

Normalized counts were converted to relative abundances and aggregated by taxonomic level. Stacked relative abundance bar plots and heat maps were created at the phylum and class levels using Tableau Desktop (version 2023.2). Bar plots and heat maps were used to visually inspect the dynamics of the predominant phyla and classes within individual samples and among study variables. Taxa with a mean relative abundance less than 1% were collapsed into an “Other” category for ease of visualization. Analysis of the Composition of Microbiomes with Bias Correction II (ANCOM-BC2) was performed to determine whether any of the bacterial classes were differentially abundant among study variables using the R packages “ANCOMBC,” “tidyverse,” “DT,” and “dplyr” (Mandal et al., 2015; Lin and Peddada, 2020, 2023). The ANCOM-BC2 model was run with a formula that included the fixed categorical covariates: day, pen change, and treatments for pooled fecal and manure pack samples. Day was indicated as the “group” variable for downstream pairwise comparisons. Other model parameters included a 10% prevalence inclusion criteria, 95% confidence level, structural zero detection, regularization factor of 5%, and bootstrap level of 100 as described by Lin and Peddada (2023). Additionally, the Holm-Bonferroni method was used to adjust *p*-values and control the false discovery rate among repeated comparisons (Holm, 1979). Model parameters reflect the recommended settings by product developers (Lin and Peddada, 2023). In the model for Day 84 samples, sample type replaced the day variable within the fixed formula and as the group variable, as previously described for pooled fecal and manure pack samples. All other model parameters were the same across sample groups. Bar charts and heatmaps were created to visualize log fold-changes in differentially abundant taxa using the R package “ggplot2” (Wickham, 2016; Lin and Peddada, 2023).

Alpha diversity was calculated using the Shannon diversity index (Shannon, 1948) and the Wilcoxon rank sum exact test using the *wilcox.test* function in the R package “vegan” was used to investigate whether study variables were associated with variation in intra-sample diversity. A linear regression model was used to investigate the individual and combined effects of day, sample type, probiotic, tylosin, and pen change on the Shannon alpha diversity metric using Stata version 17 (StataCorp, College Station, TX). Marginal mean predictions were calculated in Stata and were used to create bar plots to visually inspect diversity across samples. Weighted and unweighted UniFrac beta diversity matrices were calculated from normalized data using R packages “vegan” and “GUnifrac” to investigate dissimilarity between samples (Oksanen et al., 2022). Distance based permutational analysis of variance (PERMANOVA) function, *adonis*, was used to investigate interactions among study variables and weighted and unweighted UniFrac diversity (Anderson, 2017). Sample dispersion was characterized using the *betadisper* and *permutest* functions to confirm *adonis* significance. Weighted and unweighted UniFrac distances were used to create principal coordinates analysis (PCA) plots, which were visualized using the graphical functions of the R package “ggplot2” (Wickham, 2016).

## 3 Results

### 3.1 Description of microbiome data

For pooled fecal samples (*n* = 96), demultiplexed 16S amplicon sequence counts for forward and reverse reads ranged from 73,889 to 268,307 with an average of 142,678 and a total of 13,697,165 reads. Two pooled samples were removed from further analysis due to errors during sequencing leading to sequencing depths less than 200. These samples were both from pen 16 on day 119 of the study. Due to poor quality of reverse reads and inability of sequences to overlap during DADA2, the reverse reads were excluded from further analysis without sacrificing sequence quality. Post DADA2, reads per sample (*n* = 94) ranged from 40,452 to 177,596 with an average of 91,657 reads, which is an average retention rate of 64% (range 55−71%) of reads used for downstream analysis.

For manure pack samples (*n* = 48), demultiplexed 16S amplicon sequence counts for forward and reverse reads ranged from 36,563 to 662,653 with an average of 170,599 and a total of 8,188,750 reads. For the same reason as for pooled fecal samples, reverse reads were excluded from further analysis. Post DADA2, forward reads ranged from 27,985 to 528,372 with an average of 130,659 reads per sample, which is an average retention rate of 76% (range 66−82%) of reads used for downstream analysis.

For Day 84 samples (*n* = 48), which contains MP, DM, and PM samples from day 84 of the study prior to cattle pen change, demultiplexed 16S amplicon sequence counts for forward and reverse reads ranged from 94,495 to 662,653 with an average of 188,281 and a total of 9,037,512 reads. For the same reason as previous samples, reverse reads were excluded from further analysis. Post DADA2, there was a retention rate of 75% (range 66−83%) of reads that were used for downstream analysis. Reads per sample ranged from 68,822 to 527,232 with an average of reads 141,525 per sample.

Alpha rarefaction curves revealed sufficient plateau across all variables of interest (day, sample type, treatment, and pen change) at previously described sampling depths, indicating that sampling depth was sufficient to adequately capture taxa present. Therefore, further sampling would not meaningfully change our interpretation of the bacterial ecology present.

### 3.2 Taxonomic comparisons of bacterial communities

#### 3.2.1 Pooled fecal samples - Days 0, 84 and 119

**FIGURE 1:**
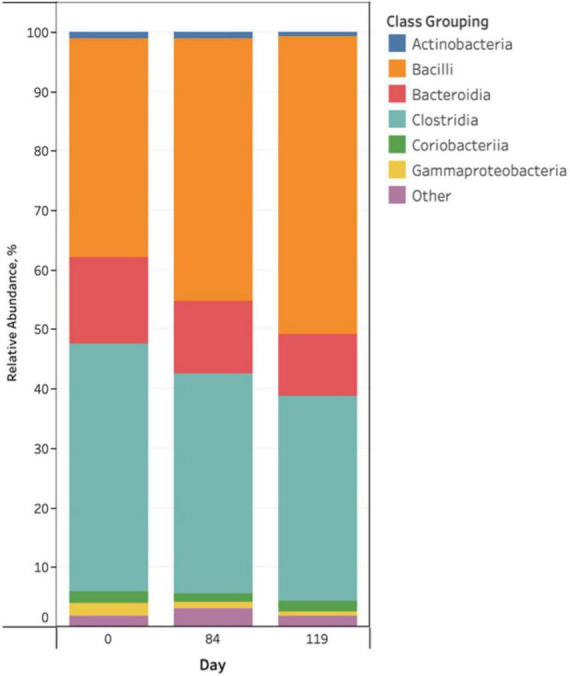
Stacked bar chart of the relative abundance of predominant bacterial classes in pooled fecal samples of beef cattle as a function of sampling day.

Taxonomic profiles indicated modest variation across sampling day (Figure 1), however, there were no visual differences as a function of pen change or treatment with antibiotics or probiotics at both the phylum and class levels. The predominant phylum was Firmicutes (69−94% abundance), followed by Bacteroidota, Actinobacteriota and Proteobacteria. The predominant phylum, Firmicutes, was characterized by two classes of bacteria, Bacilli and Clostridia, which exchanged abundance across the study days (Figure 1). Other predominant classes included: Bacteroidia, Coriobacteriia, Gammaproteobacteria, and Actinobacteria with the remainder of classes (*n* = 30) comprising less than 1% of the bacterial community (Figure 1). Rare taxa are listed in Supplemental Material. There were no obvious visual differences in bacterial taxa among treatment groups or as a function of pen change. ANCOM-BC2 was employed to detect differentially abundant phyla and classes among study variables. ANCOM-BC2 revealed that there were differentially abundant taxa for different sampling days, as a function of pen change, and in probiotic treated cattle (*P* < 0.05); however, most differences involved rare taxa (Alphaproteobacteria, Chloroflexia, Saccharimonadia, Spirochaetia, unclassified Firmicutes, Vampirivibrionia, and Verrucomicrobiae). For predominant classes, there were significant increases in Bacilli and decreases in Gammaproteobacteria between days 0 and 84 of the study. Cattle that received the probiotic treatment exhibited significantly less Gammaproteobacteria in their feces than the control group.

#### 3.2.2 Manure pack samples - Days 0, 84 and 119

**FIGURE 2:**
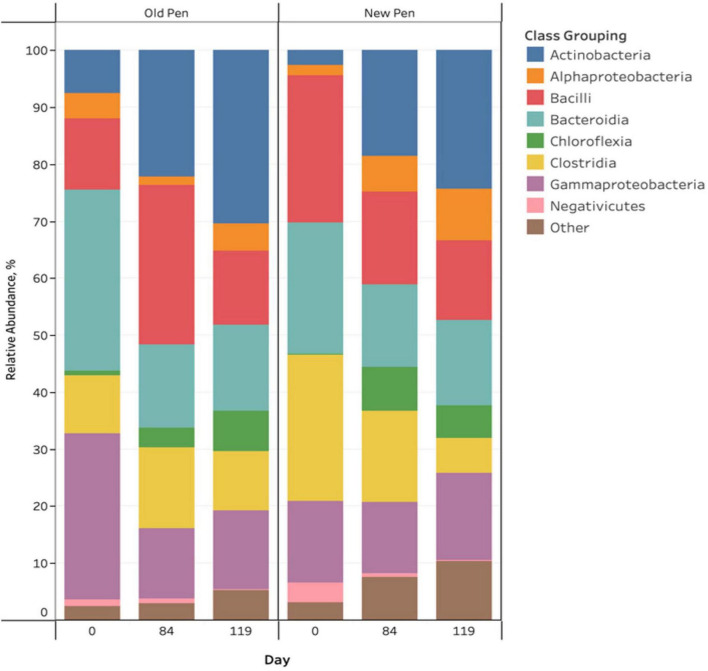
Stacked bar chart of the relative abundance of predominant bacterial classes in manure pack samples of beef cattle as a function of sampling day and old/new pen.

There were visible taxonomic changes comparing among sample days and between old and new pens for manure pack relative abundance bar plots (Figure 2). Firmicutes, Proteobacteria, Actinobacteriota, Bacteroidota and Chloroflexi were the most abundant phyla across all samples. Actinobacteria, Alphaproteobacteria, Bacilli, Bacteroidia, Chloroflexia, Clostridia, Gammaproteobacteria and Negativicutes were the predominant classes, with the remainder (*n* = 104) making up less than 1% of the bacterial community (Figure 2). Rare taxa are listed in Supplemental Material. ANCOM-BC2 revealed that there were significant differentially abundant taxa for different sampling days and in old pens compared to new pens (*P* < 0.05); however, most differences involved rare taxa (Acidimicrobiia, Anaerolineae, Armatimonadia, Bdellovibrionia, Blastocatellia, Cyanobacteria, Desulfovibrionia, Gitt-GS-136, Holophagae, KD4-96, Myxococcia, Phycisphaerae, Plantomycetes, Rhodothermia, Rubrobacteria, Saccharimonadia, Spirochaetia, Sumerlaeia, Thermoleophilia, unclassified Bacteria, unclassified Chloroflexi, unclassified Firmicutes, Vampirivibrionia, and Verrucomicrobiae). For predominant bacterial classes, there were significant increases in Actinobacteria and Chloroflexia, and significant decreases in Bacteroidia and Gammaproteobacteria between days 0 and 84 of the study. Between days 84 and 119, there was a significant decrease in Negativicutes. Old pens were shown to contain significantly more Actinobacteria than new pens. Treatments were not associated with differentially abundant taxa.

#### 3.2.3 Day 84 samples – manure pack, dried/milled, and particulate matter

**FIGURE 3:**
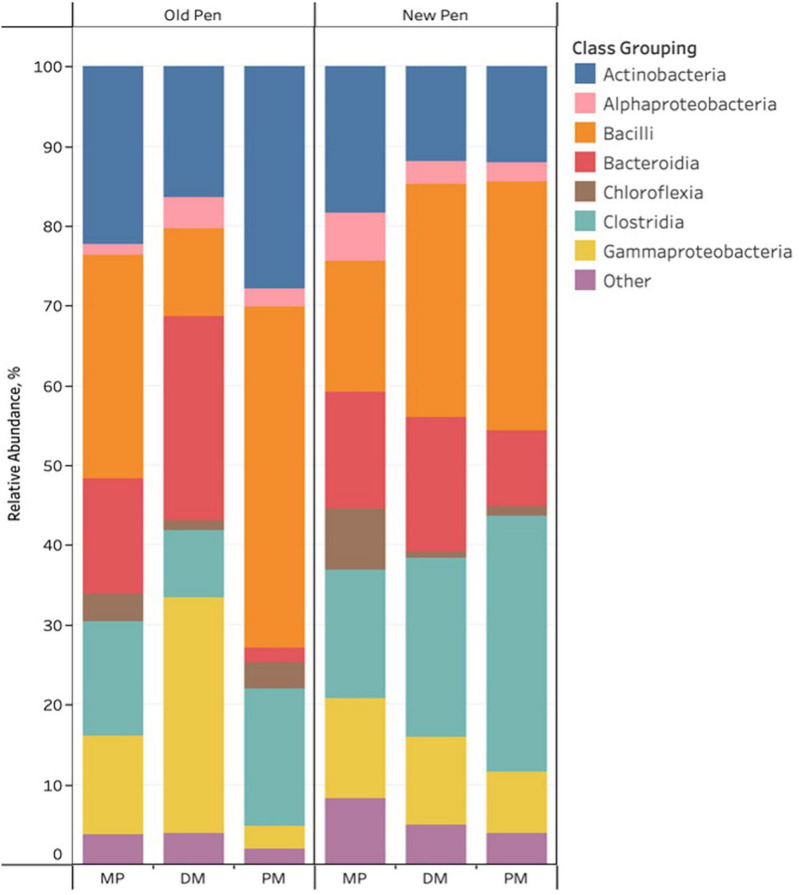
Stacked bar chart of the relative abundance of predominant bacterial classes in Day 84 manure pack (MP), dried/milled (DM) and particulate matter (PM) samples as a function of sample type and between old and new pens.

For the three different types of Day 84 samples, sample type and pen change were associated with visual differences in the relative abundance of predominant bacterial taxa. Of note, cattle were not introduced into new pens until after day 84 manure pack samples were taken. Firmicutes, Actinobacteriota, Proteobacteria, Bacteroidota and Chloroflexi were the most abundant phyla across all samples. Actinobacteria, Alphaproteobacteria, Bacilli, Bacteroidia, Chloroflexia, Clostridia, and Gammaproteobacteria were the predominant classes with the remaining classes (*n* = 107) making up less than 1% of the bacterial community (Figure 3). Rare taxa are listed in Supplemental Material. ANCOM-BC2 revealed that sample type and pen change were associated with significant differential abundance among bacterial taxa at the phylum and class levels (*P* < 0.05); however, most of the significant differences were observed in rare taxa (Acidimicrobiia, Blastocatellia, Campylobacteria, Coriobacteriia, Cyanobacteria, Cyanobacteria, Desulfovibrionia, Desulfuromonadia, Elusimicrobia, Fibrobacteria, Gitt-GS-136, Holophagae, KD4-96, Kiritimatiellae, Lentisphaeria, Myxococcia, Phycisphaerae, Plantomycetes, Polyangia, Rhodothermia, Rubrobacteria, S0134_terrestrial_group, Spirochaetia, Sumerlaeia, Syntrophobacteria, Thermoleophilia, unclassified Bacteria, unclassified Chloroflexi, unclassified Firmicutes, Vampirivibrionia, Verrucomicrobiae, and Vicinamibacteria). For predominant classes, there were significant decreases in Actinobacteria, and Chloroflexia once MP samples had been processed to DM samples. PM samples were shown to contain significantly more Actinobacteria, Bacilli, Chloroflexia, and Clostridia, and less Bacteroidia, Gammaproteobacteria and Negativicutes compared to DM samples. Old pens were shown to contain significantly more Actinobacteria than new pens. No taxa were significantly differentially abundant among treatment groups at the phylum and class levels for Day 84 samples.

### 3.3 Linear regression of Shannon alpha diversity

Linear regression was used to investigate the individual and combined effects of day, sample type, pen change, and treatment with tylosin and/or probiotics on the Shannon alpha diversity metric. Marginal mean predictions were calculated and used to create bar plots to visually inspect diversity across samples.

#### 3.3.1 Pooled fecal samples

**FIGURE 4:**
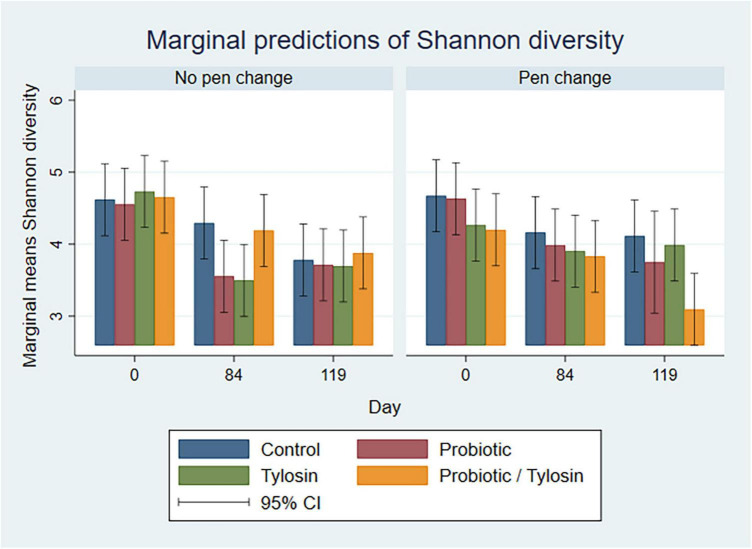
Bar plots showing linear regression marginal predictions of Shannon diversity in pooled fecal samples by treatment, sampling day and pen change.

**FIGURE 5:**
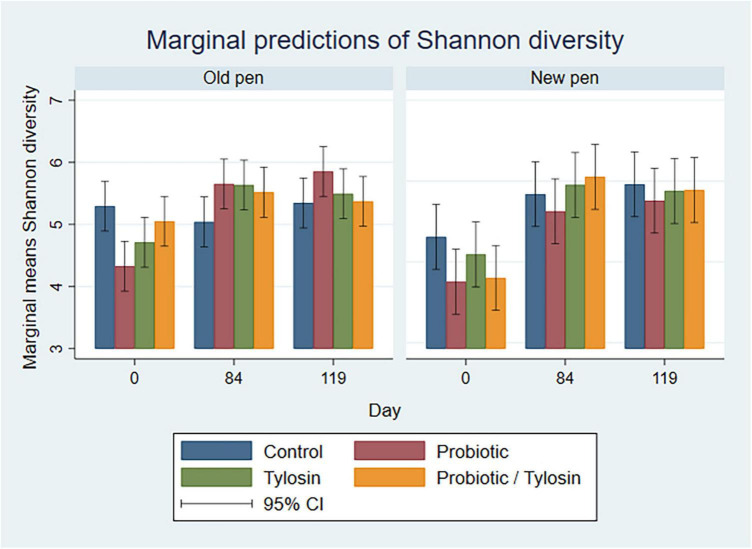
Bar plots showing linear regression marginal predictions of Shannon diversity for manure pack samples by treatment, sampling day, and between old and new pens.

In the bivariable linear regression model for pooled fecal samples, day alone accounted for approximately 31% (R-squared = 31.3%) of the variation observed in Shannon diversity. Pen change and treatment with probiotic or tylosin did not significantly influence Shannon diversity when evaluated independently. The final linear regression model for pooled fecal samples was a four-way full factorial model including day, pen change, probiotic treatment, and tylosin treatment, which accounted for approximately 48% (R-squared = 48.8%, adjusted R-squared = 32.0%) of the variation observed in the data (Figure 4). Figure 4 shows separate bar plots for cattle transferred to a new pen on day 84; however, it is important to note that cattle were co-located in their original pens until day 84 of the study. Compared to day 0, Shannon diversity was significantly decreased in the tylosin and probiotic treatment groups on days 84 and 119 (*P* < 0.05) in pooled feces of cattle that did not undergo pen change (Figure 4). Although not statistically significant, cattle transferred to a new pen on day 84 revealed a trend of decreased diversity in their feces from day 0 to 84, which is prior to their movement to new pens (Figure 4). Post pen change, pooled feces from cattle that received the combination probiotic/tylosin treatment resulted in significantly lower Shannon diversity on day 119 (*P* < 0.05), which is similar to the appearance of Shannon diversity in manure pack samples from new pens on day 0 (Figure 5).

#### 3.3.2 Manure pack samples

In the bivariable linear regression models for manure pack samples, day accounted for over 50% (R-squared = 52.5%) of the variation observed in the data, while pen change accounted for approximately 9% (R-squared = 9.3%) of the variation observed in the data. Treatment with either the probiotic or tylosin did not significantly influence Shannon diversity. The final linear regression model for manure pack samples was a four-way full factorial model including day, tylosin treatment, and probiotic treatment, which accounted for approximately 84% (R-squared = 84.5%, adjusted R-squared = 69.8%) of the variation observed in the data. Regardless of pen change, Shannon diversity significantly increased from day 0 to 84 (*P* < 0.05) in all treatment groups except the control group, then remained static for the remainder of the study (Figure 5). Day 0 samples revealed significant variation in Shannon diversity as a function of treatment although no cattle had been introduced to the pens yet (Figure 5). More specifically, the control group had significantly higher Shannon diversity than the probiotic or tylosin groups at the beginning of this study (Figure 5). There was an overall upward trend in Shannon diversity in manure pack samples across the study (Figure 5), which was opposite of what was seen in the pooled fecal samples, where diversity decreased across the study (Figure 4).

#### 3.3.3 Day 84 samples

**FIGURE 6:**
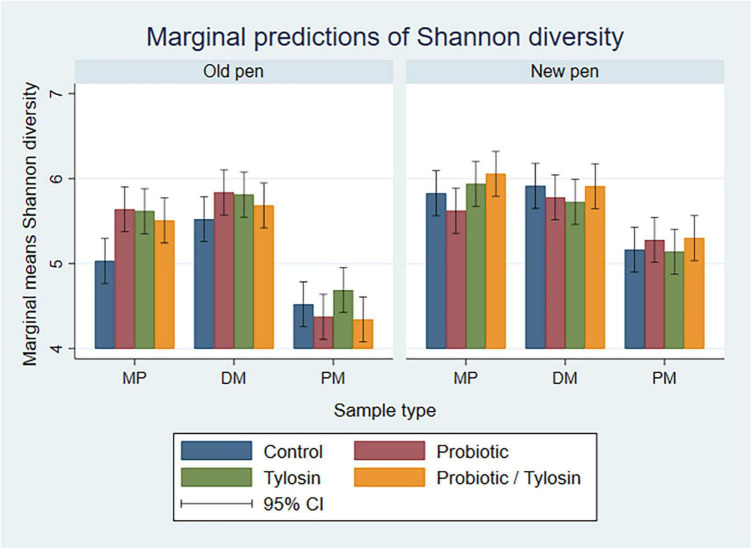
Bar plots showing linear regression marginal predictions of Shannon diversity for Day 84 manure pack (MP), dried/milled (DM) and particulate matter (PM) samples by treatment, sample type, and between old and new pens.

In the bivariable linear regression models for Day 84 samples, sample type and pen change accounted for approximately 62% (R-squared = 62.8%) and 16% (R-squared = 16.8%) of the variation observed in the data, respectively. Treatment with tylosin or the probiotic were not significant. The final linear regression model for Day 84 samples was a four-way full factorial model including sample type, pen change, tylosin treatment and probiotic treatment. This model accounted for approximately 93% (R-squared = 93.9%, adjusted R-squared = 88.0%) of the variation observed in the data. As samples were processed from MP to DM, Shannon diversity remained relatively static, whereas, PM samples were associated with a significantly lower (*P* < 0.05) Shannon diversity, especially in old pens (*P* < 0.05) (Figure 6).

### 3.4 Weighted and unweighted UniFrac analysis of beta diversity

Weighted and unweighted UniFrac measures were calculated using the distance based PERMANOVA function to investigate interactions between study variables and weighted and unweighted UniFrac diversity. PCA plots were generated from UniFrac dissimilarity matrices to visually inspect sample distribution.

#### 3.4.1 Pooled fecal samples

**FIGURE 7:**
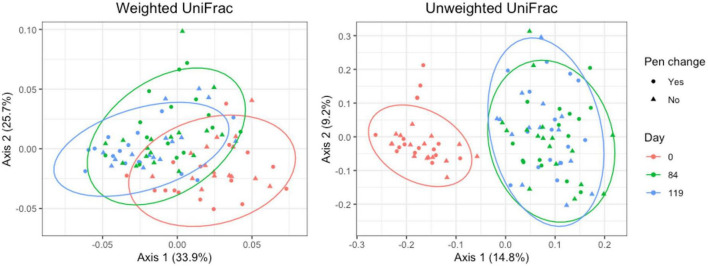
Principal coordinates analysis (PCA) plots of pooled fecal samples showing the differences in beta diversity among sampling days and between old and new pens using weighted and unweighted UniFrac dissimilarity matrices.

For pooled fecal samples, weighted and unweighted UniFrac distances varied significantly by day (Adonis *P* = 0.001 and *P* = 0.001, respectively) and pen change (Adonis *P* = 0.039 and *P* = 0.001, respectively). Both weighted and unweighted UniFrac plots revealed clustering with stronger clustering patterns when using unweighted UniFrac distance (Figure 7). Samples from day 84 and 119 overlap, indicating that the taxa present and their relative abundances are similar between the two days. There were no significant associations between pen change or treatment for beta diversity measures. Based on the top two axes, weighted and unweighted PCA plots accounted for 60 and 24% of the variation in the data, respectively.

#### 3.4.2 Manure pack samples

**FIGURE 8:**
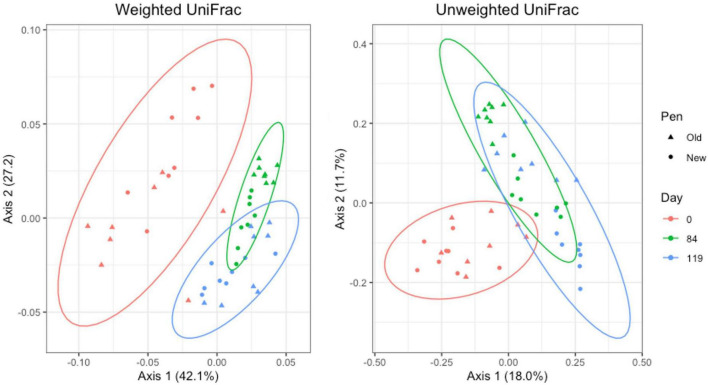
Principal coordinates analysis (PCA) plots of manure pack samples showing the differences in beta diversity among sampling days and between old and new pens using weighted and unweighted UniFrac dissimilarity matrices.

For manure pack samples, weighted and unweighted UniFrac distances differed significantly by day (Adonis *P* = 0.001 and *P* = 0.001, respectively) and pen change (Adonis *P* = 0.016 and *P* = 0.001, respectively). PCA plots showed a clear separation of day 0 samples from day 84 and 119. Similar to the pooled fecal samples, day 84 and 119 manure pack samples overlap (Figure 8). Within day 0 samples, pen change did not appear to influence UniFrac diversity. On days 84 and 119, samples from old pens clustered separately from new pens, indicating that the taxa present and their relative abundances are distinct between the two groups. Treatment group was not associated with differences in weighted or unweighted UniFrac diversity. Based on the top two axes, weighted and unweighted PCA accounted for 70 and 30% of the variation in the data, respectively.

#### 3.4.3 Day 84 samples

**FIGURE 9:**
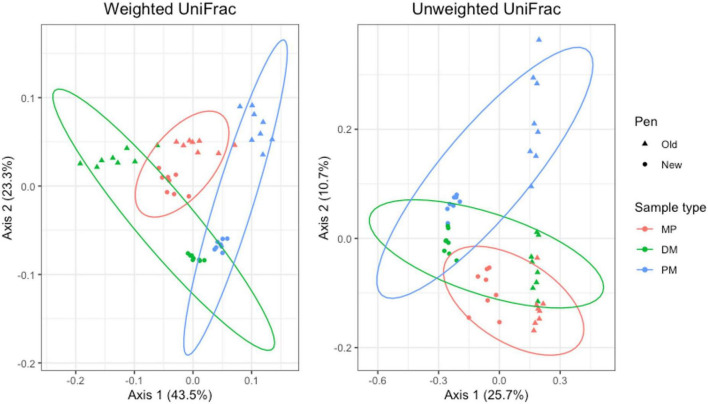
Principal coordinates analysis (PCA) plots of Day 84 manure pack (MP), dried/milled (DM) and particulate matter (PM) samples showing the differences in beta diversity among sample types and between old and new pens using weighted and unweighted UniFrac dissimilarity matrices.

**FIGURE 10:**
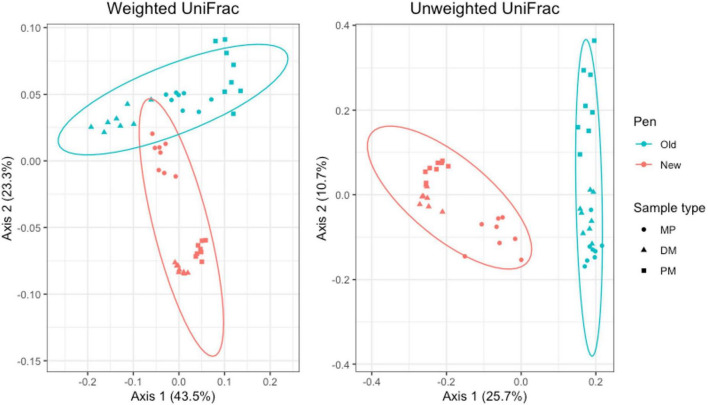
Principal coordinates analysis (PCA) plots of Day 84 manure pack (MP), dried/milled (DM) and particulate matter (PM) samples showing the differences in beta diversity between old and new pens and among sample types using weighted and unweighted UniFrac dissimilarity matrices.

For Day 84 samples, weighted and unweighted UniFrac distances varied significantly by sample type (Adonis *P* = 0.001 and *P* < 0.014, respectively) and pen change (Adonis *P* = 0.001 and *P* = 0.001, respectively). PCA plots show independent clustering of samples as a function of sample type (Figure 9) and pen change (Figure 10). MP samples from old and new pens clustered more closely together than to the DM or PM samples from the same old or new pen. Contrastingly, DM and PM samples from old pens were more closely related to each other than to their corresponding sample type in new pens. In this way, PM originating from old pens contained significant differences in the taxa present/absent and their relative abundances than PM originating from new pens. Moreover, PM samples were significantly different than the MP they originated from. Treatment group was not associated with variation in beta diversity between samples. Based on the top two axes, weighted and unweighted PCA plots account for 67 and 36% of the variation in the data, respectively (Figures 9, 10).

## 4 Discussion

This study utilized 16S rRNA amplicon sequencing to characterize the bacterial community structure as a function of antibiotics, probiotics, and pen environment change on the bacterial communities present in cattle feces, the feedyard environment, and airborne particulate matter. It is widely accepted that antibiotic administration to high-risk cattle successfully reduces the occurrence of diseases that impact their performance and health; however, increased scrutiny around the use of antibiotics in food animals has elucidated the need for alternatives that promote animal health without contributing to the emergence and persistence of AMR in animals and their associated feedyard environment. Moreover, as research continues to highlight the public health risk associated with airborne particulate matter originating from food animal production systems (Smit, 2012; Luiken et al., 2020; Urso et al., 2021), it is important to characterize the effects of antibiotics and antibiotic alternatives on the bacteria present in particulate matter that has the potential to transmit to surrounding areas.

Similar to previous findings, our study did not reveal a significant impact on diversity or bacterial communities present within cattle feces or their environment as a function of treatment with antibiotics (Noyes et al., 2016; Doster et al., 2018). Contrastingly, other researchers have observed a significantly lower Shannon diversity in cattle treated with tylosin compared to control animals (Weinroth et al., 2019); however, there were no alterations in bacterial communities observed. Differences in geographic location, study methodologies, sequencing modalities, and bioinformatic analyses likely contribute to the lack of congruency among studies investigating the effects of antibiotics and antibiotic alternatives on animal health, performance characteristics, morbidity, and microbial ecology. To better understand changes in the microbial communities during the finishing period in beef cattle, we evaluated the microbiomes within feces, manure pack and particulate matter within the same feedyard facility.

Day of the study and pen change were associated with significant differences in biodiversity metrics (alpha and beta diversity) across all sample types; however, this was not always reflected by changes in the predominant taxa. Interestingly, day 0 manure pack samples revealed significant differences in Shannon diversity among treatment groups although no cattle were present at that time. These differences were most likely the result of residual effects from the first trial of the study by Murray et al. (2020), and represent the reality of the feedyard infrastructure where previous cohorts of finishing cattle affect subsequent groups of cattle. Additionally, we observed that the Shannon diversity in pooled fecal samples changed to reflect the diversity of the manure pack in the pens to which the cattle were introduced. This is seen in old pens on day 84 and new pens on day 119, both of which were the first fecal sampling day after cattle were introduced into the pens. Additionally, manure pack samples revealed significantly higher Shannon diversity than pooled fecal samples on days 84 and 119 of the study. These findings support previous research which showed that freshly voided feces exhibited significantly lower Shannon diversity than that of cattle feedyard soil (Noyes et al., 2016) and soil collected adjacent to a beef cattle feedyard facility (Zaheer et al., 2019). Further work is needed to understand the complexities and adaptations of microbial community dynamics between cattle and the feedyard environment.

The probiotic used in our study has been shown to exhibit “faecal-environmental-oral cycling” as described by (Murray et al., 2020); however, we did not observe an increase in Bacilli, the class to which the probiotic is classified. Instead, we observed that probiotic treated cattle feces contained significantly less Gammaproteobacteria, which contains many genera of importance to human health including the foodborne bacteria *Escherichia coli* and *Salmonella spp.* We suspect that the administration of the probiotic may have caused a shift in the microbiome that promoted a decrease in Gammaproteobacteria; however, the implications of this are not fully understood at this time. Increased resolution into the specific orders or genera that are influenced by probiotic administration may shed light into whether aforementioned medically relevant organisms are similarly decreased by this antibiotic alternative. One group of researchers suggest that the phylum Proteobacteria (to which Gammaproteobacteria is classified) may play a role in nitrogen cycling and levels of nitrous oxide emissions from the feedyard environment (Waldrip et al., 2022). In this way, the probiotic used in this study may play a role in the way nitrogen is utilized by the bacterial communities in cattle feces; which may in turn affect animal performance and health, although that was not investigated in this study. Otherwise, there were no significant differences in biodiversity or predominant bacterial taxa within pooled feces, manure pack, or particulate matter as a function of treatment with the probiotic.

We identified differentially abundant classes in each group of samples, however, some of these classes comprised less than 1% of the bacterial community. Rare taxa have been shown to have the potential to rapidly expand and cause meaningful shifts to their associated bacterial communities (Shade et al., 2014); however, in our study there were no meaningful shifts to the community as a function of these rare taxa. Additional work is needed to understand the implications of taxa found to be statistically differentially abundant while remaining less than 1% abundant in the community. Deeper resolution into taxonomic changes using 16S rRNA amplicon sequencing in conjunction with more comprehensive sequencing technologies, such as shotgun sequencing, transcriptomics, and full metagenome characterization may give a more robust understanding of the effects of probiotics and antibiotics within cattle feces, the feedyard environment, and airborne PM.

Multiple studies have quantified bacterial taxa and antimicrobial resistance determinants in airborne particulate matter around cattle feedyards (McEachran et al., 2015; Wooten et al., 2019); however, to our knowledge, this is the first study to compare bacterial communities within fecal, environmental manure pack and airborne particulate matter samples within the same feedyard facility. We modeled MP desiccation to airborne PM to characterize the microbial changes experienced in the cattle feedyard environment over time. Interestingly, the predominant bacterial classes within PM samples more closely resembled the structure of fecal samples than the MP samples from which they originated. This suggests that as desiccation occurs, there may be significant changes to the bacterial communities to a more Firmicutes-dominated population by the time particulate matter is created. The phylum Firmicutes contains many classes of organisms that are known to be drought tolerant, such as Bacilli and Clostridia, both of which were shown to be higher in PM and fecal samples than environmental MP in our study. These findings agree with a previous study that found airborne PM collected downwind from a feedyard facility contained phyla and genera most closely associated with cattle feces; however, they were not able to collect pen level samples for comparison (McEachran et al., 2015). Although not well characterized in beef cattle, researchers investigating the microbiomes within poultry and pig farms revealed that the microbial communities present within PM correlated significantly with the fecal microbiome (Luiken et al., 2020). Further work is needed to determine if the artificially desiccated PM in this study is representative of naturally occurring PM in the feedyard environment. As our study has shown distinct bacterial communities and biodiversity metrics between pooled feces, manure pack, and particulate matter samples, we suggest that it may not be sufficient to use fecal or environmental samples as a proxy to characterize the public health risk associated with particulate matter from food animal production systems.

## 5 Conclusions

In an effort to combat AMR in food animal production systems, many alternatives to antibiotics have been investigated to promote animal health and performance characteristics of beef cattle during the finishing period. Microbial probiotics, fermentation products and environmental management changes have been suggested as strategies to mitigate AMR in the presence of cattle on antibiotics. Our study revealed varying environmental microbial communities between old and new pens, however, after the introduction of cattle into new pens, these differences were less evident. Although there were changes in cattle feces diversity that reflected the initial environmental conditions, the movement of cattle into new pens did not meaningfully affect the microbial communities within pooled fecal samples, specifically at the end of the finishing period when an effect would be most impactful for public health. Administration of an *Enterococcus faecium/Saccharomyces cerevisiae* probiotic may have promoted a shift in the fecal microbiome leading to a decrease in Gammaproteobacteria; however, treatment was otherwise not associated with changes to the fecal, manure pack or particulate matter bacterial communities. Our study was the first to characterize and compare microbial communities within feces, manure pack and airborne particulate matter from the same location, revealing significantly different bacterial populations. In this way, additional sampling efforts of particulate matter from food animal production systems should be performed in future studies that aim to characterize potential microbial exposures to feedyard personnel and nearby communities. Determining bacterial community dynamics is an essential step in understanding the effects of antibiotics and their alternatives on food animal production systems and inferring public health risk associated with airborne particulate matter.

## Data availability statement

The datasets presented in this study can be found in online repositories. The names of the repository/repositories and accession number(s) can be found below: https://www.ncbi.nlm.nih.gov/, PRJNA595617.

## Ethics statement

The animal study was approved by the Agriculture Animal Care and Use Committee (AACUC AUP #2015-026A). The study was conducted in accordance with the local legislation and institutional requirements.

## Author contributions

AS: Conceptualization, Data curation, Formal Analysis, Methodology, Visualization, Writing – original draft, Writing – review & editing. SM: Data curation, Methodology, Writing – review & editing, Conceptualization. JV: Data curation, Supervision, Writing – review & editing, Conceptualization, Methodology. BA: Data curation, Methodology, Writing – review & editing. KB: Data curation, Methodology, Writing – review & editing. JS: Methodology, Writing – review & editing, Funding acquisition. HS: Data curation, Formal Analysis, Funding acquisition, Project administration, Supervision, Writing – review & editing. KN: Data curation, Formal Analysis, Methodology, Project administration, Supervision, Writing – review & editing.
