# Corrigendum: Comparative microbiome analysis of beef cattle, the feedyard environment, and airborne particulate matter as a function of probiotic and antibiotic use, and change in pen environment

**DOI:** 10.3389/fmicb.2024.1422959

**Published:** 2024-05-08

**Authors:** A. H. Strickland, S. A. Murray, J. Vinasco, B. W. Auvermann, K. J. Bush, J. E. Sawyer, H. M. Scott, K. N. Norman

**Affiliations:** ^1^Department of Veterinary Integrative Biosciences, Texas A&M University, College Station, TX, United States; ^2^Department of Veterinary Pathobiology, Texas A&M University, College Station, TX, United States; ^3^Texas A&M AgriLife Research and Extension Center at Amarillo, Amarillo, TX, United States; ^4^Department of Animal Sciences, Texas A&M University, College Station, TX, United States

**Keywords:** environmental microbiome, fecal microbiome, particulate matter, antibiotic alternatives, antimicrobial resistance (AMR)

In the published article, there was an error in the Funding statement. The authors did not include the NIH T32 project number (T32 OD011083) with the original version of the NIH T32 acknowledgement. The correct Funding statement appears below.

